# Dr. Edward Duguid Ritchie

**Published:** 1912-08-03

**Authors:** 


### Obituary.

DR. EDWARD DUGUID RITCHIE has died at his residence near Southampton, at the age of fifty-two, after a long illness. His medical education was pursued at St. Thomas's Hospital and at Cambridge. He qualified in 1885, and took his Cambridge degrees, including the M.A., in the following year. At St. Thomas's Hospital he held the resident appointments of house physician and house surgeon. Dr. Ritchie was always attracted by country life, and took up private practice first in Sevenoaks and then near Camberley, Surrey. Eventually he removed to Hampshire, where he practised in Blackwater and later in Chandler's Ford until overtaken by ill-health. As a country practitioner he was not only successful professionally, but also played the part of the country gentleman to the great benefit of the community.

